# Regional effects of endocannabinoid, BDNF and FGF receptor signalling on neuroblast motility and guidance along the rostral migratory stream

**DOI:** 10.1016/j.mcn.2014.12.001

**Published:** 2015-01

**Authors:** Ya Zhou, Madeleine J. Oudin, Sangeetha Gajendra, Martina Sonego, Katarzyna Falenta, Gareth Williams, Giovanna Lalli, Patrick Doherty

**Affiliations:** Wolfson Centre for Age-Related Diseases, King's College London, London SE1 1UL, United Kingdom

**Keywords:** Neuroblast migration rostral migratory stream, Endocannabinoid, BDNF, FGF

## Abstract

During development and after birth neural stem cells in the subventricular zone (SVZ) generate neuroblasts that migrate along the rostral migratory stream (RMS) to populate the olfactory bulb (OB) with neurons. Multiple factors promote neuroblast migration, but the contribution that many of these make to guidance within the intact RMS is not known. In the present study we have characterised in detail how endocannabinoid (eCB), BDNF and FGF receptor (FGFR) signalling regulates motility and guidance, and also determined whether any of these receptors operate in a regionally restricted manner. We used *in vivo* electroporation in postnatal mice to fluorescently label neuroblasts, and live cell imaging to detail their migratory properties. Cannabinoid receptor antagonists rendered neuroblasts less mobile, and when they did move guidance was lost. Similar results were obtained when eCB synthesis was blocked with diacylglycerol lipase (DAGL) inhibitors, and importantly eCB function is required for directed migration at both ends of the RMS. Likewise, inhibition of BDNF signalling disrupted motility and guidance in a similar manner along the entire RMS. In contrast, altering FGFR signalling inhibits motility and perturbs guidance, but only at the beginning of the stream. Inhibition of FGFR signalling *in vivo* also reduces the length of the leading process on migratory neuroblasts in a graded manner along the RMS. These results provide evidence for a guidance function for all three of the above receptor systems in the intact RMS, but show that FGFR signalling is unique as it is required in a regionally specific manner.

## Highlights

• Live imaging is used to characterise neuroblast migration in the intact RMS.
• Endocannabinoids are required for motility and guidance throughout the RMS.
• BDNF regulates guidance in a similar manner.
• FGFR signalling is also required for guidance, but only at the beginning of the stream.
• FGFR regulates neuroblasts morphology in a graded manner along the RMS *in vivo*.

## 1 Introduction

During development neural stem cells in the subventricular zone (SVZ) give rise to neuroblasts that migrate along the rostral migratory stream (RMS) to the olfactory bulb (OB) where they differentiate into new neurons (Alvarez-Buylla et al., 2000). This long-range pathway persists into adulthood in most mammals, but is transient in the human brain, disappearing about two years after birth (Sanai et al., 2011). However, neurogenesis is maintained into old age in the human SVZ, with new neurons appearing in the adjacent striatum throughout life (Ernst et al., 2014). In some circumstances SVZ-derived neuroblasts can migrate to injured areas of the brain where they might limit damage and restore function (Arvidsson et al., 2002; Christie and Turnley, 2012; Young et al., 2011). However, the molecular pathways regulating neuroblast migration in health and disease remain incompletely understood.

The migration of neuroblasts along the RMS can be regulated by many factors, including guidance and cell adhesion molecules and growth factors (Lalli, 2014). We have recently shown that the endocannabinoid (eCB) system regulates proliferation of neural stem cells in the SVZ (Gao et al., 2010; Goncalves et al., 2008) and the migration of their progeny neuroblasts out of RMS explants (Oudin et al., 2011). However, the polarity of the stream is lost in explant cultures making it difficult to determine if eCBs, and other factors, simply have a motogenic function or whether they also regulate guidance. A better understanding of migration dynamics can be obtained by real time imaging of neuroblasts within the relatively intact RMS in brain slice cultures (Nam et al., 2007). These can be used to identify factors that affect motility and local guidance, and to determine if pathways operate in a regionally specific manner.

Here we focussed our attention on eCB, FGF and BDNF signalling as there is compelling evidence for all three regulating neuroblast migration from RMS explant cultures with limited or no evidence describing their effect on neuroblast dynamics and guidance along the different areas of the intact stream (Bagley and Belluscio, 2010; Chiaramello et al., 2007; Garcia-Gonzalez et al., 2010; Oudin et al., 2011; Snapyan et al., 2009). To clarify how these pathways control neuroblast migration in the post-natal mouse brain, we have used *in vivo* electroporation and live cell imaging to fluorescently label SVZ-derived neuroblasts and analyse their migration along the RMS (Sonego et al., 2013b). Our results show that eCB and BDNF signalling are required for motility and guidance throughout the RMS. In contrast, altering FGFR signalling affects motility and guidance at the beginning of the RMS, but has no significant effect towards the end of the stream. Inhibition of FGFR signalling *in vivo* also has a spatially restricted influence on neuroblasts, affecting their morphology at the beginning, but not in the end of the RMS. These results suggest that eCB and BDNF signalling are required to guide neuroblasts along the entire stream, whereas the FGFR operates in a regionally restricted manner, likely responding to a gradient of FGF-2 emanating from the SVZ.

## 2 Results

### 2.1 eCB signalling is required for directed cell migration within the RMS

**Fig. 1:**
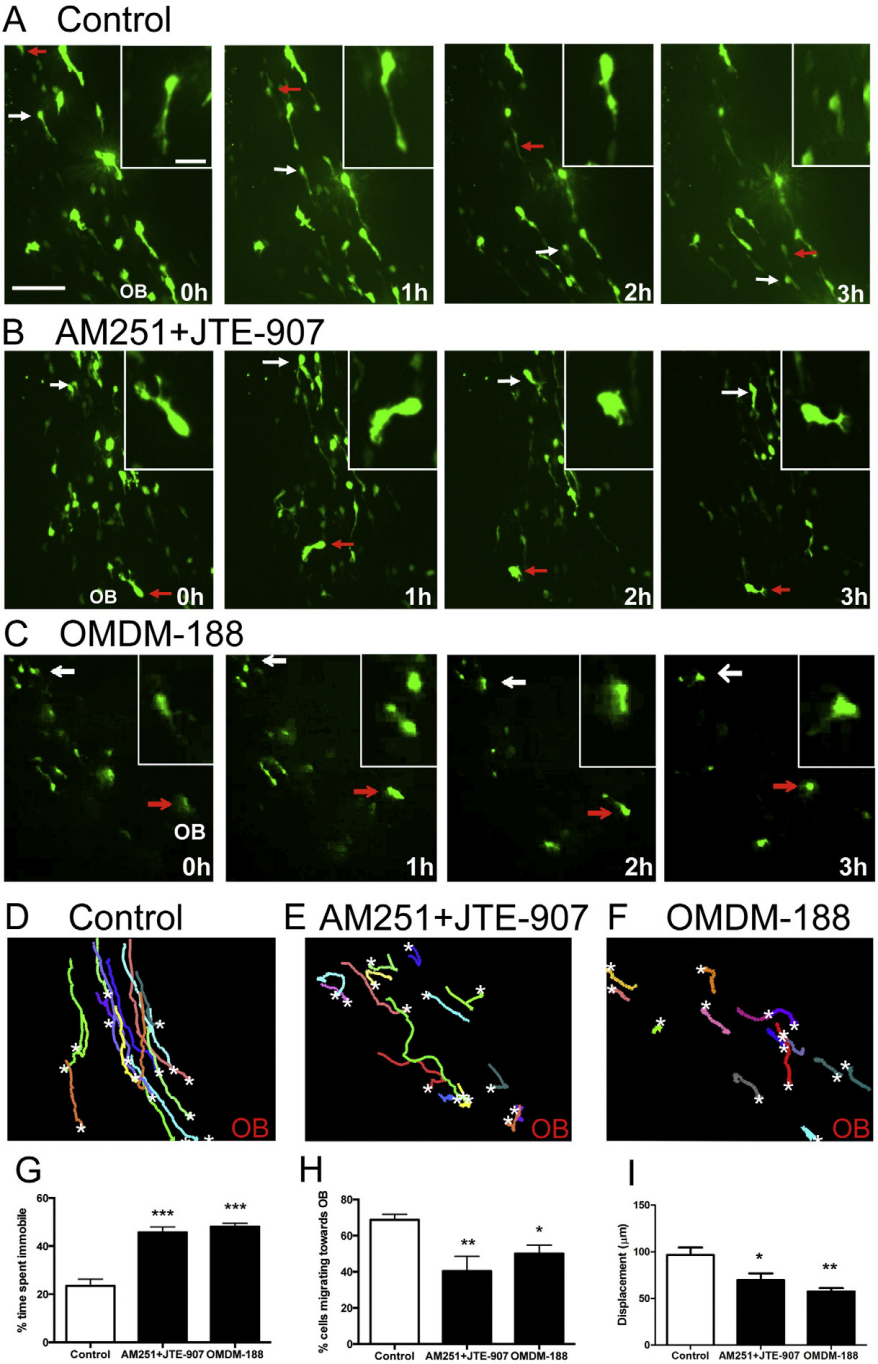
eCB  signalling regulates cell migration in the RMS. Sagittal mouse brain slices with GFP-labelled neuroblasts were prepared 5–7 days after *in vivo* postnatal electroporation of P2 mice with pCX-EGFP, cultured with vehicle or drugs for 2 h and subsequently imaged for 3 h in the same medium. Time-lapse movies made from the descending arm of the RMS in slices treated with different drugs targeting the eCB system (the CB1/2 antagonists AM251 + JTE-907 or the DAGL inhibitor OMDM188, all at 1 μM) were analysed using Volocity. Representative pictures of slices treated with vehicle (A) or the CB1/2 antagonists AM251 + JTE-907 (1 μM each) (B) or OMDM-188 (1 μM) (C) are shown. Arrows follow two neuroblasts in each frame. Insets show magnifications of the neuroblast indicated by the white arrows (A) and (C) or red arrow (B). Representative migratory tracks of 15 cells over 3 h from a control (D), a CB1/2 antagonist-treated (E) or a DAGL inhibitor-treated brain slice (F). White stars mark the tracking end point of each cell. The OB label shows the location of the olfactory bulb in each brain slice. Cells with decreased eCB signalling spent more time immobile (G). Incubation with CB antagonists or DAGLs inhibitors also significantly decreased the percentage of neuroblasts migrating towards the OB (H) and the overall cell displacement (I). Graphs show mean ± s.e.m. (n = 7 brain slices for each condition, ~ 15–30 cells analysed per slice); *p < 0.05, **p < 0.01, ***p < 0.001. Bars, 35 μm for (A–B), 10 μm for insets. (For interpretation of the references to color in this figure legend, the reader is referred to the web version of this article.)

**Fig. 2:**
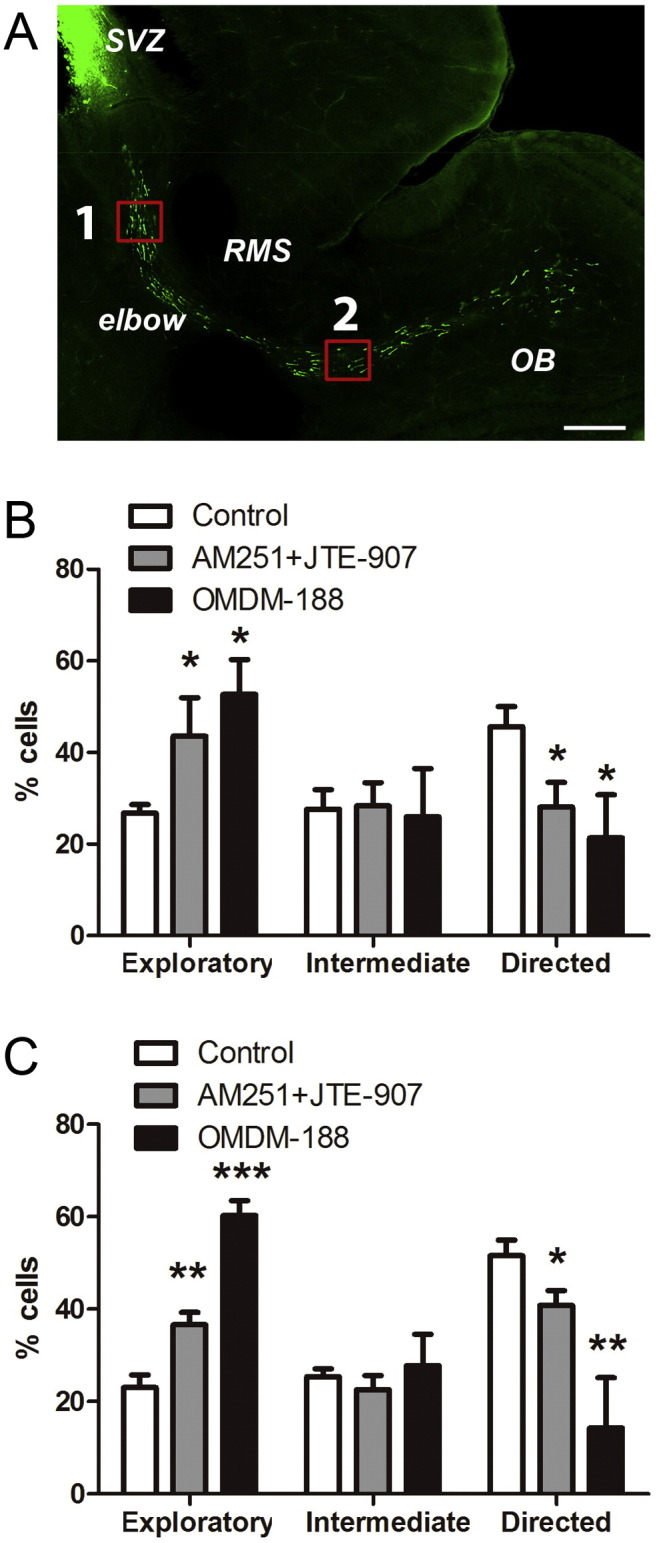
Inhibiting eCB signalling affects neuroblast directionality along the RMS. Five to seven days after *in vivo* electroporation of pCX-EGFP in P2 mice, brain slices were cultured with vehicle or drugs for 2 h followed by time-lapse imaging for 3 h in the same medium. (A) A typical sagittal brain slice showing labelled neuroblasts. The two red rectangular boxes labelled 1 and 2 indicate the imaging sites in the beginning (caudal) and end (rostral) of RMS, respectively. Incubation with CB1 and CB2 antagonists (AM251 + JTE-907, 1 μM each), or with OMDM-188 (1 μM), significantly increased the percentage of exploratory neuroblasts at the expense of directed neuroblasts in both regions of the RMS (B, caudal RMS; C, rostral RMS). Graphs show mean ± s.e.m. (n = 6 brain slices for each condition, ~ 15–30 cells analysed per slice); *p < 0.05, **p < 0.01, ***p < 0.001. Bar, 400 μm.

eCB signalling promotes SVZ neuroblast migration *in vitro*, and a single 24 hour treatment with either CB1 or CB2 antagonists decreases the length of the leading process of RMS neuroblasts *in vivo* (Oudin et al., 2011). Here, we used time-lapse imaging of GFP-labelled neuroblasts in brain slice cultures to determine if eCB is simply motogenic or whether it plays a role in guiding neuroblasts along the RMS. Slices were equilibrated for 2 h in control medium or medium containing a combination of CB1/2 antagonists (AM251 and JTE-907, both at 1 μM) before being imaged for 3 h. Initial imaging was focused on the descending arm of the RMS before the “elbow” region (region 1 in Fig. 2A). Individual neuroblasts were then tracked and migration analysed (see methods for details). Representative images of migrating neuroblasts from brain slices treated with the vehicle control, CB1/2 antagonist (Fig. 1A,B) and the DAGL inhibitor (Fig. 1C) are shown. The white and red arrows highlight the position of two neuroblasts in each condition over a 3 hour period. Control neuroblasts migrated over longer distances towards the OB compared to neuroblasts in slices treated with the CB1/2 antagonists (Fig. 1A, B) or the DAGL inhibitor (Fig. 1 A–C). In addition, control cells displayed a predominant unipolar morphology usually oriented towards the OB, while cells treated with cannabinoid antagonists often displayed branched processes extending in all directions (Fig. 1A–C insets).

The migration tracks of individual neuroblasts from representative slices are shown in Fig. 1D–F. Under control conditions neuroblasts tended to follow a similar path towards the OB (Fig. 1D; Supplementary material Movie 1). Remarkably, when the CB receptors were inhibited, the cells could still move, but migration was clearly less directed (Fig. 1E, Supplementary material Movie 2). A detailed statistical analysis of cell migration dynamics in these experiments is summarised in Fig. 1G–I. In control slices, neuroblasts were immobile (nucleus moved less than 2 μm) ~ 25% of their time, but this increased to almost 45% (p < 0.001) when CB1/2 receptors were inhibited (Fig. 1G). Interestingly, when the cells did move in the presence of the CB1/2 antagonists they did so at a similar speed to control cells (60.5 ± 3.5 and 58.4 ± 3.4 μm/h, respectively). In agreement with a recent study (Bagley and Belluscio, 2010), when imaging over a 3 hour period, ~ 70% of neuroblasts show net migration towards the OB in control medium (Fig. 1H), and this was significantly reduced by treatment with the cannabinoid receptor antagonists to around 40% (Fig. 1H). Total cell displacement was also substantially reduced (Fig. 1I). The same results were obtained by blocking the activity of the DAGL enzymes that are responsible for the synthesis of the endogenous cannabinoid receptor ligand 2-AG with OMDM-188 (Ortar et al., 2008) (1 μM) (Fig. 1C, F and G–I) or THL (data not shown).

The migration tracks of individual neuroblasts from representative slices are shown in Fig. 1D–F. Under control conditions neuroblasts tended to follow a similar path towards the OB (Fig. 1D; Supplementary material Movie 1). Remarkably, when the CB receptors were inhibited, the cells could still move, but migration was clearly less directed (Fig. 1E, Supplementary material Movie 2). A detailed statistical analysis of cell migration dynamics in these experiments is summarised in Fig. 1G–I. In control slices, neuroblasts were immobile (nucleus moved less than 2 μm) ~ 25% of their time, but this increased to almost 45% (p < 0.001) when CB1/2 receptors were inhibited (Fig. 1G). Interestingly, when the cells did move in the presence of the CB1/2 antagonists they did so at a similar speed to control cells (60.5 ± 3.5 and 58.4 ± 3.4 μm/h, respectively). In agreement with a recent study (Bagley and Belluscio, 2010), when imaging over a 3 hour period, ~ 70% of neuroblasts show net migration towards the OB in control medium (Fig. 1H), and this was significantly reduced by treatment with the cannabinoid receptor antagonists to around 40% (Fig. 1H). Total cell displacement was also substantially reduced (Fig. 1I). The same results were obtained by blocking the activity of the DAGL enzymes that are responsible for the synthesis of the endogenous cannabinoid receptor ligand 2-AG with OMDM-188 (Ortar et al., 2008) (1 μM) (Fig. 1C, F and G–I) or THL (data not shown).

To look for regional differences within the RMS, we analysed populations of neuroblasts sampled towards the beginning or end of the RMS as indexed by region 1 and region 2 in Fig. 2A. Here we monitored the directionality of neuroblast migration by calculating the “meandering” index (MI), *i.e.* the ratio between net displacement and total distance covered over the 3 hour time period. According to this ratio, neuroblast movement can be classified as *exploratory* (ratio < 0.4), *directed* (ratio > 0.6), or *intermediate* (0.4–0.6) (Nam et al., 2007). In the RMS, close to the SVZ (Fig. 2A), treatment with CB receptor antagonists resulted in a near two-fold increase in the number of exploratory neuroblasts at the expense of a near two-fold decrease in the number of cells migrating in a directed manner (Fig. 2B). Very similar results were observed when neuroblasts were analysed in the RMS close to the OB (Fig. 2C). Inhibition of DAGL activity with OMDM-188 (1 μM) led to similar results in both regions of the RMS (Fig. 2B, C). Thus, we can conclude that DAGL-dependent eCB signalling is required for neuroblast motility and guidance within the RMS and that this pathway operates all along the RMS.

### 2.2 BDNF signalling is required for directed cell migration within the RMS

BDNF has a motogenic effect on neuroblasts in RMS explant cultures (Chiaramello et al., 2007). The role of BDNF signalling in regulating neuroblast motility and directionality along the entire intact stream is less clear as an antibody reported to block BDNF increases neuroblast motility without disrupting guidance (Bagley and Belluscio, 2010), while another report showed that inhibiting BDNF signalling in the adult mouse brain impairs RMS neuroblast migration and directionality (Snapyan et al., 2009). We have revisited this question by determining the effect of TrkB-Fc on neuroblast motility and guidance along different regions of the stream; this reagent binds BDNF and prevents it from interacting with TrkB and the p75NTR (Binder et al., 1999; Snapyan et al., 2009).

**Fig. 3:**
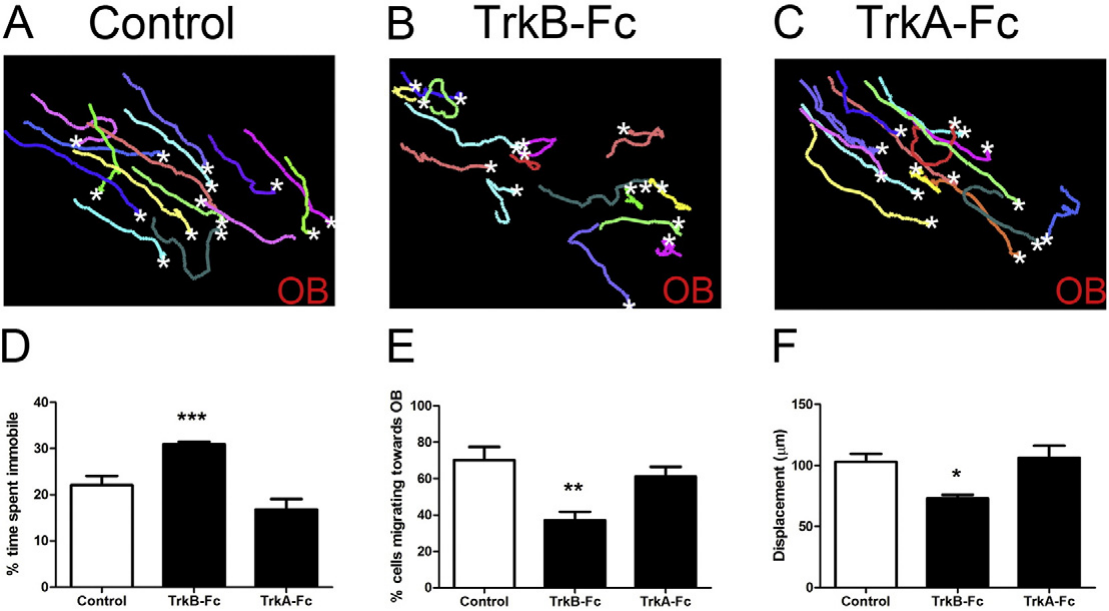
BDNF signalling is required for efficient polarised neuroblast migration. Time-lapse movies made from the descending arm of the RMS in slices with GFP-labelled neuroblasts incubated with TrkB-Fc (blocking BDNF signalling) or TrkA-Fc as a control (all at 1 μg/ml) were analysed using Volocity. Representative migratory tracks of 15 cells over a 3 hour filming period from a control (A), a TrkB-Fc (B) or a TrkA-Fc-treated brain slice (C). White stars mark the end point of each migration track. OB shows the location of the olfactory bulb in each brain slice. Blocking BDNF signalling increased the percentage of time spent immobile by neuroblasts (D), significantly decreased the percentage of cells migrating towards the OB (E) and the overall cell displacement (F). Incubation with TrkA-Fc did not have any significant effect on all the measured parameters (D–F). Graphs show mean ± s.e.m. (n = 6–8 brain slices for each condition, ~ 15–30 cells analysed per slice); *p < 0.05, **p < 0.01, ***p < 0.001.

Visual inspection of the migration tracks in the descending arm of the RMS demonstrates that neuroblasts can still migrate in the presence of TrkB-Fc (Fig. 3B, Supplementary material Movie 3), however tracks were shorter than control and their directionality was clearly disrupted (Fig. 3A,B). Detailed statistical analysis showed that in the presence of TrkB-Fc neuroblasts were immobile for longer periods of time (Fig. 3D), with a substantial reduction from ~ 70 to ~ 40% in the percentage of cells moving towards the OB (Fig. 3E). Moreover, we observed a significant decrease in the net displacement of the neuroblast population (Fig. 3F). There was a relative small effect on the average speed of TrkB-Fc-treated cells (control 61.2 ± 1.8 μm/h, and TrkB-Fc 51.6 ± 2.7 μm/h, P < 0.05). In order to control for non-specific effects of the Fc-chimera, we also treated cultures with a TrkA-Fc blocking NGF but not BDNF signalling (Binder et al., 1999) at the same concentration. This had no significant effect on any of the measured parameters including average speed of migration (Fig. 3C–F).

Visual inspection of the migration tracks in the descending arm of the RMS demonstrates that neuroblasts can still migrate in the presence of TrkB-Fc (Fig. 3B, Supplementary material Movie 3), however tracks were shorter than control and their directionality was clearly disrupted (Fig. 3A,B). Detailed statistical analysis showed that in the presence of TrkB-Fc neuroblasts were immobile for longer periods of time (Fig. 3D), with a substantial reduction from ~ 70 to ~ 40% in the percentage of cells moving towards the OB (Fig. 3E). Moreover, we observed a significant decrease in the net displacement of the neuroblast population (Fig. 3F). There was a relative small effect on the average speed of TrkB-Fc-treated cells (control 61.2 ± 1.8 μm/h, and TrkB-Fc 51.6 ± 2.7 μm/h, P < 0.05). In order to control for non-specific effects of the Fc-chimera, we also treated cultures with a TrkA-Fc blocking NGF but not BDNF signalling (Binder et al., 1999) at the same concentration. This had no significant effect on any of the measured parameters including average speed of migration (Fig. 3C–F).

**Fig. 4:**
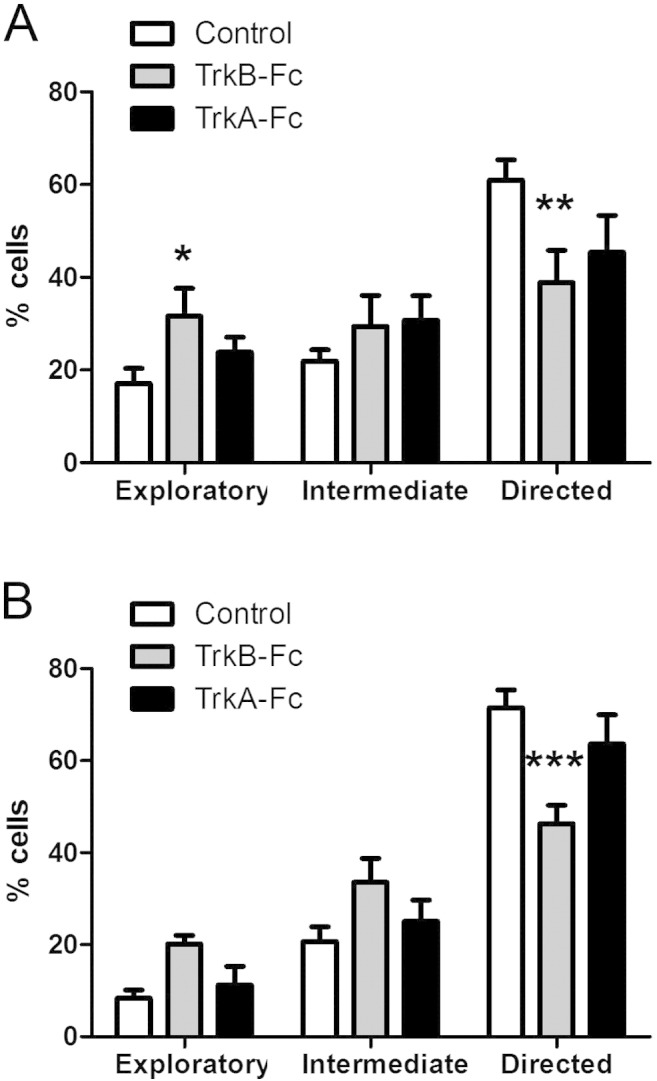
Inhibiting BDNF signalling affects neuroblast directionality along the RMS. Five days after *in vivo* electroporation of pCX-EGFP in P2 mice, brain slices were cultured with vehicle, TrkA-Fc or TrkB-Fc (1 μg/ml) for 2 h followed by time-lapse imaging for 3 h in the same medium. Neuroblast migration was filmed and analysed in the beginning and end of RMS (regions 1 and 2 in Fig. 2A). Incubation with TrkB-Fc significantly increased the percentage of exploratory neuroblasts at the expense of directed neuroblasts towards the beginning (A) and end of the RMS (B). Incubation with TrkA-Fc did not have any significant effect compared to control slices. Graphs show mean ± s.e.m. (n = 6–8 brain slices for each condition, ~ 15–30 cells analysed per slice); *p < 0.05, **p < 0.01, ***p < 0.001.

To quantitatively examine the effect of inhibiting BDNF signalling on the directionality of neuroblast migration towards the beginning and end of the RMS, we again calculated the meandering index in region 1 and 2 (see above). The most conspicuous observation was a highly significant reduction in the number of neuroblasts showing directed migration in presence of TrkB-Fc, and importantly this was apparent at both ends of the stream (Fig. 4A,B). Again, TrkA-Fc had no significant effect. Thus we can conclude that BDNF signalling is required for neuroblast motility and guidance within the RMS, and that this pathway operates all along the stream.

### 2.3 FGFR function is required for directed migration at the beginning of the RMS

**Fig. 5:**
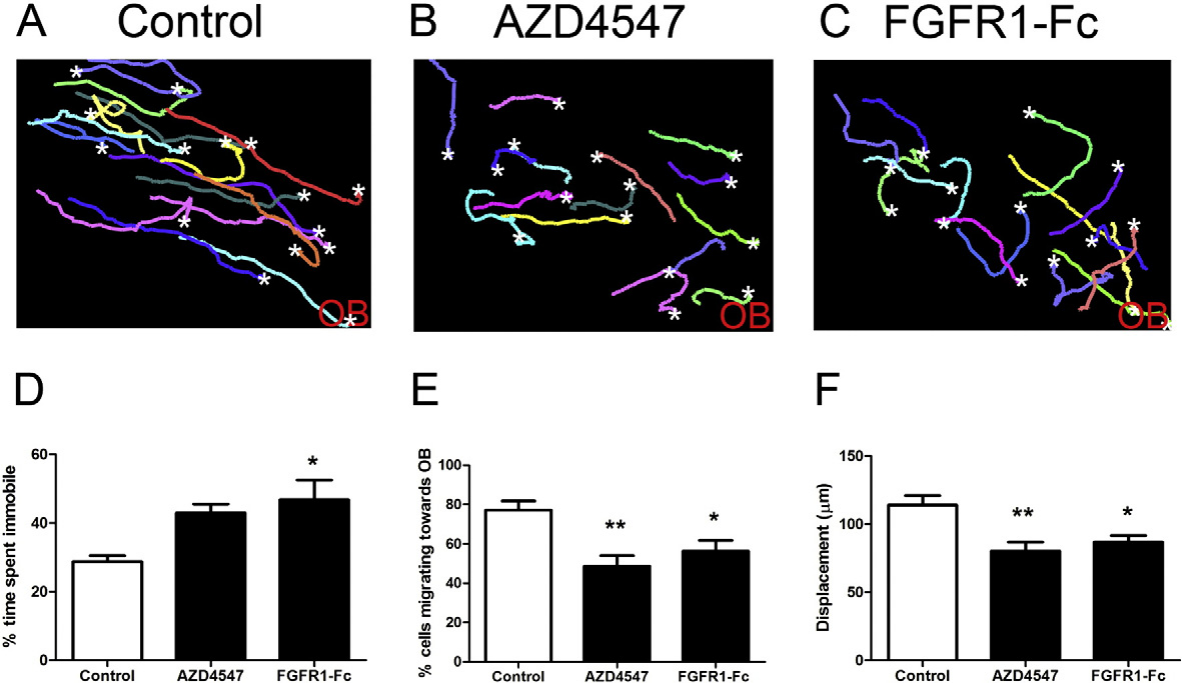
FGFR signalling regulates neuroblast migration in the RMS. Time-lapse movies of GFP-labelled neuroblasts in the descending arm of the RMS from slices treated with different drugs targeting the FGFR signalling system (1 μM AZD4547 or 1 μg/ml FGFR1-Fc) were analysed using Volocity. Representative migratory tracks of 15 cells over a 3 hour filming period from a control (A), an AZD4547 (B) or a FGFR1-Fc (C) -treated brain slice. White stars mark the end point of each migration track, OB indicates the location of the olfactory bulb. Inhibiting FGF signalling increased the percentage of time spent immobile by neuroblasts (D), while significantly decreasing the percentage of cells migrating towards the OB (E) and the overall cell displacement (F). Graphs show mean ± s.e.m. (n = 6–7 brain slices for each condition, ~ 15–30 cells analysed per slice); *p < 0.05, **p < 0.01.

SVZ neuroblasts express at least one FGFR and FGF-2 has a motogenic rather than chemotropic effect on neuroblast migration from RMS explant cultures (Garcia-Gonzalez et al., 2010). To test the role of the FGFR in the intact RMS, we initially treated slice cultures with the highly selective FGFR inhibitor AZD4547 (1 μM) (Gavine et al., 2012). Initial imaging was focused on the descending arm of the RMS before the “elbow” region (region 1 in Fig. 2A). Visual inspection of neuroblast migration tracks showed a very clear reduction in distance and a loss of guidance when the FGFR inhibitor was present (Fig. 5B, Supplementary material Movie 4). Detailed analysis showed this to be associated with the neuroblasts tending to spend more time immobile (Fig. 5D) with significant reductions in the number of neuroblasts migrating towards the OB (~ 75% to 45%, Fig. 5E) and a parallel reduction in total displacement of the population (Fig. 5F). Again when the cells did move, they moved at a speed similar to control neuroblasts (57.8 ± 1.5 μm/h for the control, and 53.6 ± 3.4 μm/h for the AZD4547-treated slices). Similar results were obtained with PD173074, an independent FGFR inhibitor (Mohammadi et al., 1998) (data not shown). In order to substantiate the above results we also treated slice cultures with an FGFR1-Fc chimera (1 μg/ml) to trap any FGFR1 ligands present within the RMS. The results observed with this agent did not differ from the results obtained with the small molecule receptor inhibitors (Fig. 5 C–F).

SVZ neuroblasts express at least one FGFR and FGF-2 has a motogenic rather than chemotropic effect on neuroblast migration from RMS explant cultures (Garcia-Gonzalez et al., 2010). To test the role of the FGFR in the intact RMS, we initially treated slice cultures with the highly selective FGFR inhibitor AZD4547 (1 μM) (Gavine et al., 2012). Initial imaging was focused on the descending arm of the RMS before the “elbow” region (region 1 in Fig. 2A). Visual inspection of neuroblast migration tracks showed a very clear reduction in distance and a loss of guidance when the FGFR inhibitor was present (Fig. 5B, Supplementary material Movie 4). Detailed analysis showed this to be associated with the neuroblasts tending to spend more time immobile (Fig. 5D) with significant reductions in the number of neuroblasts migrating towards the OB (~ 75% to 45%, Fig. 5E) and a parallel reduction in total displacement of the population (Fig. 5F). Again when the cells did move, they moved at a speed similar to control neuroblasts (57.8 ± 1.5 μm/h for the control, and 53.6 ± 3.4 μm/h for the AZD4547-treated slices). Similar results were obtained with PD173074, an independent FGFR inhibitor (Mohammadi et al., 1998) (data not shown). In order to substantiate the above results we also treated slice cultures with an FGFR1-Fc chimera (1 μg/ml) to trap any FGFR1 ligands present within the RMS. The results observed with this agent did not differ from the results obtained with the small molecule receptor inhibitors (Fig. 5 C–F).

**Fig. 6:**
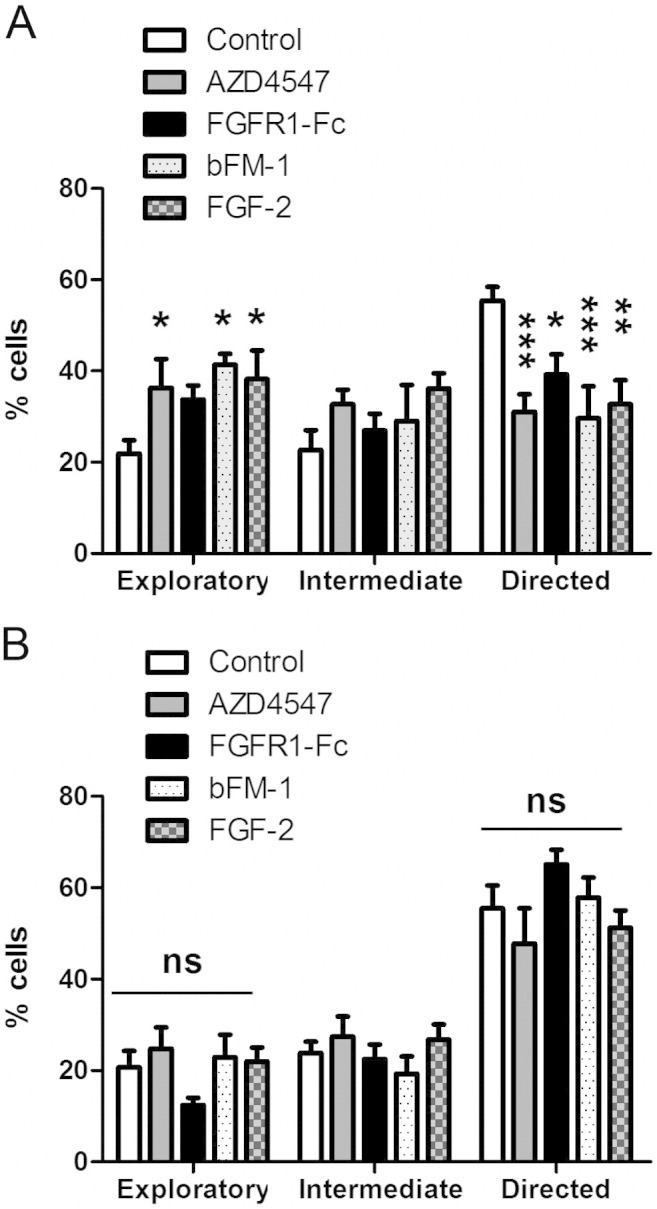
Targeting FGF signalling affects neuroblast migration in the beginning but not in the end of RMS. Acute mouse brain slices containing GFP-labelled neuroblasts were prepared 5–7 days after *in vivo* electroporation of pCX-EGFP, cultured with vehicle or drugs for 2 h and imaged for 3 h in the same medium. Targeting the FGF signalling system using two distinct FGFR1 inhibitors (AZD4547 1 μM, or FGFR1-Fc 1 μg/ml), a FGF-2 neutralising antibody (bFM-1, 10 μg/ml), or FGF-2 (50 ng/ml) decreased the percentage of cells migrating in a directed fashion and increased the amount of exploratory neuroblasts in the beginning (A) but not in the end of RMS (B). Graphs show mean ± s.e.m. (n = 5–8 brain slices for each condition, ~ 15–30 cells analysed per slice); *p < 0.05, **p < 0.01, ***p < 0.001.

We next asked if there were regional differences in FGFR signalling by extending our analysis to the proximal and distal RMS. For cells sampled close to the beginning of the stream, treatment with the FGFR inhibitor AZD4547 significantly increased the number of neuroblasts showing exploratory migration at the expense of those showing directed migration (Fig. 6A). Again, a very similar result was seen with PD173074 (not shown) and with the FGFR1-Fc, and also with an antibody that specifically inhibits FGF-2 (bFM-1, 10 μg/ml), suggesting that this is probably the ligand driving the FGFR response (Fig. 6A). To test whether a gradient of endogenous FGF-2 might be playing an instructive role in migration as suggested by others (Garcia-Gonzalez et al., 2010), we treated the slice cultures with a relatively high concentration of exogenous FGF-2 (50 μg/ml) to “flatten” any gradient. This also disrupted guidance in a similar manner as the other treatments, significantly increasing the number of exploratory cells at the expense of the number of directed cells (Fig. 6A, Supplementary material Movie 5).

We next asked if there were regional differences in FGFR signalling by extending our analysis to the proximal and distal RMS. For cells sampled close to the beginning of the stream, treatment with the FGFR inhibitor AZD4547 significantly increased the number of neuroblasts showing exploratory migration at the expense of those showing directed migration (Fig. 6A). Again, a very similar result was seen with PD173074 (not shown) and with the FGFR1-Fc, and also with an antibody that specifically inhibits FGF-2 (bFM-1, 10 μg/ml), suggesting that this is probably the ligand driving the FGFR response (Fig. 6A). To test whether a gradient of endogenous FGF-2 might be playing an instructive role in migration as suggested by others (Garcia-Gonzalez et al., 2010), we treated the slice cultures with a relatively high concentration of exogenous FGF-2 (50 μg/ml) to “flatten” any gradient. This also disrupted guidance in a similar manner as the other treatments, significantly increasing the number of exploratory cells at the expense of the number of directed cells (Fig. 6A, Supplementary material Movie 5).

Remarkably, whereas all of the above treatments significantly reduced directed migration at the beginning of the RMS, they had no significant impact on this towards the end of the stream (Fig. 6B). Also, whereas the total population displacement was reduced at the beginning of the stream (113.8 ± 7.1 μm for control, 80.2 ± 6.6 μm for AZD4547, 86.8 ± 4.8 μm for FGFR1-Fc, 76.1 ± 8.0 μm for bFM-1, 76.2 ± 4.8 μm for FGF-2, p < 0.01) this was unaffected at the end of the stream by all four treatments (105.1 ± 7.5 μm for control, 110.5 ± 8.6 μm for AZD4547, 123.7 ± 5.8 μm for FGFR1-Fc, 103.6 ± 4.7 μm for bFM-1, 112.1 ± 4.6 μm for FGF-2, p > 0.05). Thus, we can conclude that FGF-2/FGFR signalling is required for neuroblast motility and guidance within the RMS, but that in contrast to eCB and BDNF signalling, this pathway only operates towards the beginning of the RMS.

### 2.4 FGFR and eCB signalling pathways function independently in RMS explant cultures

**Fig. 7:**
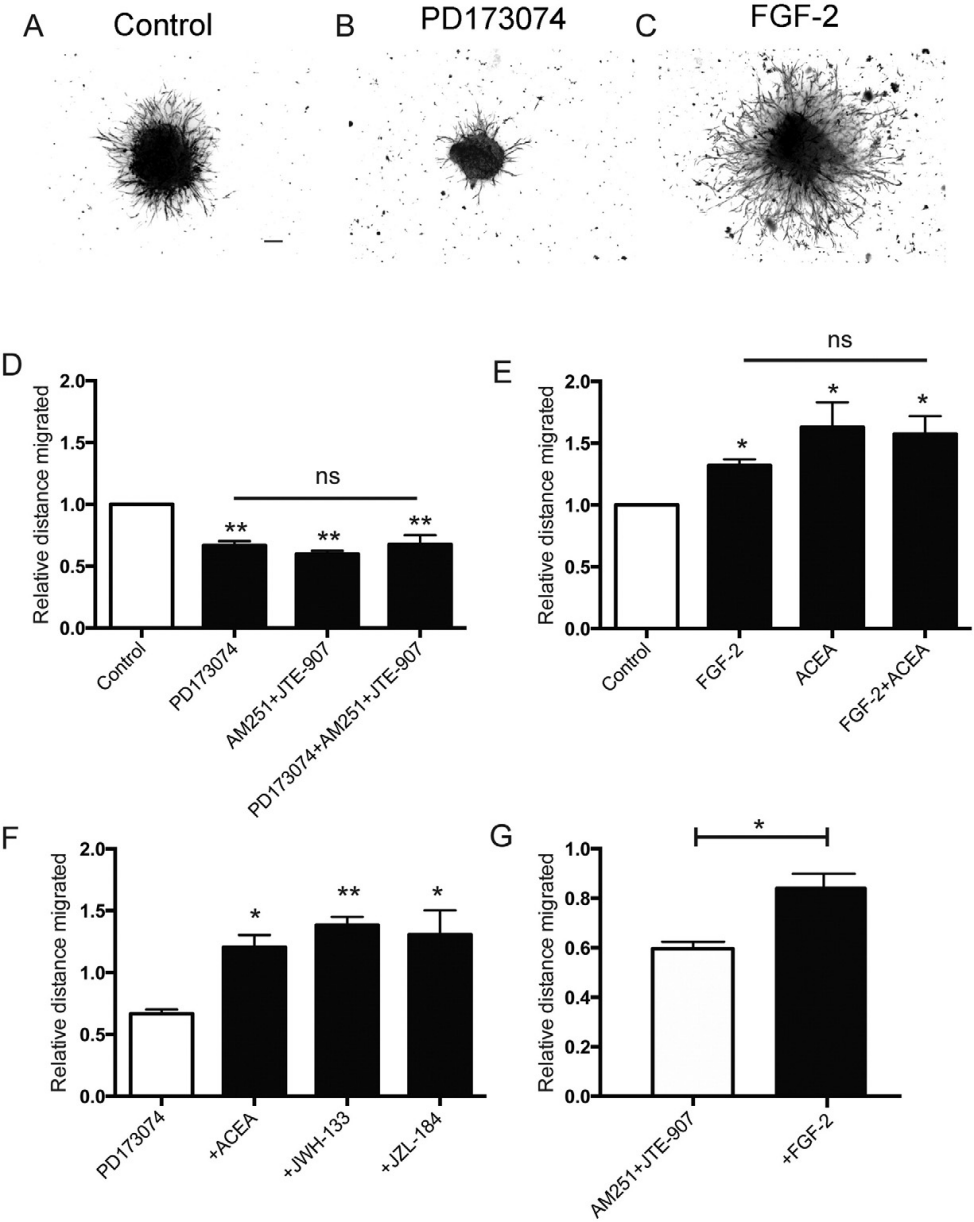
FGF and eCB signalling can operate independently in culture. RMS explants isolated from P5–P8 mouse brains were embedded in Matrigel and left to migrate for either 6 h or 24 h before fixation. Representative pictures of explants stained with phalloidin treated with vehicle (A), 1 μM of the FGFR inhibitor PD173074, (B) or 2 ng/ml FGF-2 (C) taken 24 h after embedding. In the 6 h assay, treatment with 1 μM PD173074 significantly decreased migration to the same extent observed after incubation with the CB1/2 antagonists AM251 and JTE-907 (0.5 μM each) (D). When FGFR and CB receptors were inhibited simultaneously, there was no further significant decrease in migration (D). In the 24 h assay FGF-2 (2 ng/ml) significantly promoted migration to the same level observed after incubation with the CB1 agonist ACEA (0.5 μM), and treatment with both did not cause any additive effect on migration (E). In the 24 h assay the CB1 agonist ACEA, the CB2 agonist JWH-133 and the MAGL inhibitor JZL-184 (all at 0.5 μM) could all still significantly stimulate migration in the presence of the FGFR inhibitor PD173074 (1 μM) (F). In the presence of the CB receptor antagonists AM251 and JTE-907 (0.5 μM each), FGF-2 (2 ng/ml) still induced a significant migratory response in the 24 h assay. Graphs show mean ± SEM (n = 4); *p < 0.05, **p < 0.01, ***p < 0.001. Bar, 100 μm for (A–C).

CB1 and/or CB2 antagonists inhibit the migration of neuroblasts out of RMS explant cultures, suggesting the presence of an endogenous cannabinoid tone (Oudin et al., 2011). We were interested to know if the FGF and BDNF pathways are also active in explant cultures. The FGFR inhibitor PD173074 (1 μM) significantly reduced migration out of RMS explants to the same degree as seen with inhibition of CB1/2 receptors with AM251 and JTE-907 (both at 0.5 μM), and interestingly the effects of inhibiting the FGFR and eCB receptors were not additive (Fig. 7A, B and D). In contrast, K252a (1 μM) a potent inhibitor of the Trk receptor family, including TrkB (Tapley et al., 1992), had no significant effect on migration out of the explant cultures (value measured in presence of the drug was 88 ± 7% of the control, mean ± s.e.m, n = 3).

CB1 and CB2 agonists, and a MAGL inhibitor which stimulates eCB receptors by increasing 2-arachidonylglycerol (2-AG) levels, all promote neuroblasts migration form RMS explants (Oudin et al., 2011). In the present study we see a very similar response to FGF-2 (2 ng/ml) (Fig. 7C), and this was not additive to a CB1 agonist (ACEA, 0.5 μM) response (Fig. 7E). Importantly, a CB1 agonist (ACEA), a CB2 agonist (JWH-133) and a MAGL inhibitor (JZL-184) (all at 0.5 μM) all stimulated a robust migratory response in the presence of the FGFR inhibitor (Fig. 7F) to a level not dissimilar to the maximal response seen in the absence of the FGFR inhibitor (compare Fig. 7E and F). Similarly, FGF-2 significantly stimulated neuroblast migration in the presence of a CB1/CB2 receptor block (Fig. 7G), however this response was muted compared to the effect caused by FGF-2 alone in control cultures (compare Fig. 2G and E). These data suggest that the FGFR and eCB receptors work mainly *via* independent signalling pathways, but that the FGF-2 response might be to some extent dependent on the eCB pathway.

### 2.5 FGFR signalling regulates neuroblast morphology in the RMS *in vivo*

**Fig. 8:**
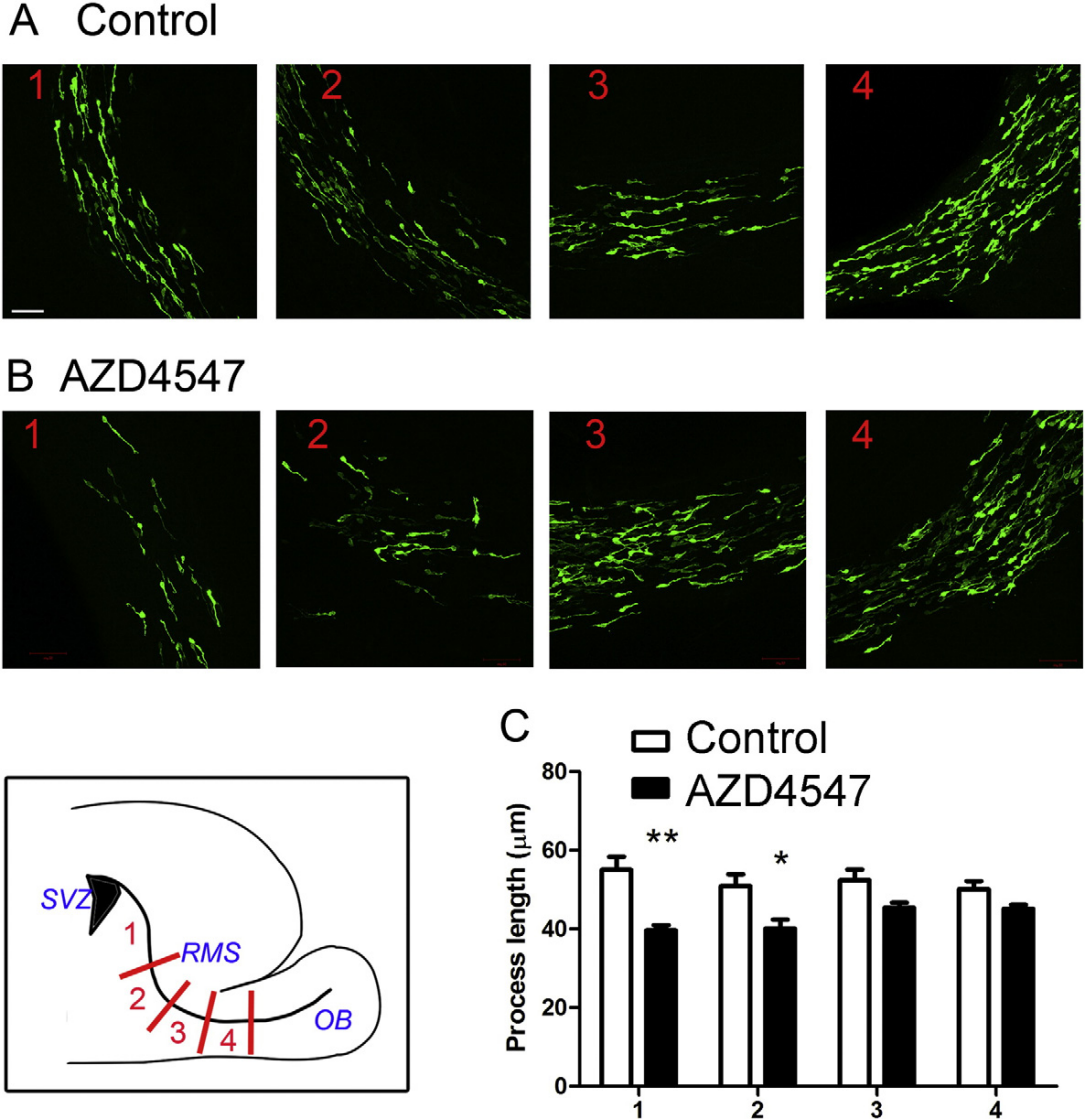
Signalling through FGFR regulates the morphology of migrating neuroblasts *in vivo*. P2 mouse pups were electroporated with pCX-EGFP and 5 days later treated with the FGFR inhibitor AZD4547 (12.5 mg/kg I.P., two doses with 12 hour interval). After 24 h, brains were fixed, sliced and stained for GFP. Representative pictures of migrating neuroblasts in animals treated with vehicle (A) and AZD4547 (B) in 4 different regions along the RMS (labelled 1–4 as depicted in the cartoon). Inhibiting FGFR signalling significantly decreased the process length of migrating neuroblasts only in regions 1 and 2 of the RMS (C). Graphs show mean ± s.e.m. (n = 3–4 animals for each condition, 6 consecutive slices were analysed per brain, ~ 100–200 cells analysed per region); *p < 0.05, **p < 0.01. Bar, 50 μm for (A–B).

We have previously shown changes in neuroblast morphology in the RMS following treatment of living animals with CB1 and CB2 antagonists (Oudin et al., 2011). In the present study we treated mice with the selective FGFR inhibitor AZD4547 to determine if this is sufficient to alter neuroblast morphology along the RMS *in vivo*. In brief, 2 day-old mouse pups were electroporated to label SVZ-derived neuroblasts with GFP and returned to their mothers. Five days later the animals were treated with a double (12 h interval) I.P. administration of AZD4547 (12.5 mg/kg), sacrificed after 24 h, and their brains processed for neuroblast morphology analysis in four defined regions along the RMS (see cartoon in Fig. 8) as previously described (Oudin et al., 2011). We observed a very clear gradated response, with cells towards the beginning of the stream showing a highly significant decrease in leading process length of ~ 25%, and cells at the distal end of the stream showing no response (Fig. 8). This provides evidence for FGFR signalling regulating neuroblast morphology *in vivo*, and supports the conclusion made with slice cultures pointing to regional differences in FGFR signalling within the RMS.

## 3 Discussion

Neuroblasts migrate in highly characteristic chains within the RMS, with overall movement directed towards the OB. Removal of the OB has no effect on guidance, suggesting that this is determined by factors emanating from the SVZ and/or synthesised locally within the stream (Bagley and Belluscio, 2010). Many molecules can regulate neuroblast migration, but most of these have been shown to primarily affect motility rather than regional guidance, and examples include GABA (Bolteus and Bordey, 2004) and metalloproteases (Bovetti et al., 2007). In the present study we have used live cell imaging of neuroblasts in intact brain slices to determine if eCB, BDNF and FGF receptor signalling have roles beyond their motogenic effects established on cultured cells (Chiaramello et al., 2007; Garcia-Gonzalez et al., 2010; Oudin et al., 2011). Our results show that all three can regulate both neuroblast motility and local guidance, but whereas the eCB and BDNF do so throughout the stream, the FGFR operates in a regionally restricted manner. We will consider each in turn, before addressing some key unresolved issues.

The *in vivo* administration of CB1 or CB2 antagonists leads to altered neuroblast morphology within the RMS, and inhibition of the migration of RMS neuroblasts in culture, with the individual contributions of each receptor generally being similar (Oudin et al., 2011). Detailed transcriptional profiling also shows that the CB1 and CB2 receptors are coupled to the same signalling pathways in migratory progenitor cells (Sutterlin et al., 2013). In the present study we used live imaging to determine if and how eCB signalling regulates migration in the RMS within intact brain slices. In this context, we inhibited eCB signalling by blocking the synthesis of 2-AG, or by the combined use of highly selective CB1/CB2 antagonists. Both treatments resulted in the cells spending longer periods of time in an immobile state, however when the cells moved they migrated at the similar speed as control cells. Interestingly, the migratory neuroblasts followed a random rather than directed pathway with the overall net effect being a substantial reduction in the number of cells migrating towards the OB. The pathway operates throughout the RMS, with similar perturbations seen at both ends of the stream.

Our previous studies show co-expression of the DAGLs and of the CB1 receptor in the same SVZ-derived neuroblasts, suggesting a largely autocrine signalling pathway (Oudin et al., 2011). Migratory progenitor cells and neuroblasts respond to CB2 agonists and antagonists in a manner consistent with this receptor also functioning in an autocrine manner (Oudin et al., 2011; Sutterlin et al., 2013). Nevertheless, given that neuroblasts migrate over each other in chains, it is also possible that 2-AG might exert paracrine effects on neighbouring cells, with this type of “short-range” signalling recently shown to be capable of regulating axonal guidance (Keimpema et al., 2013). However, 2-AG is unlikely to be a long-range chemoattractant in the RMS as it has a short half-life and does not diffuse over long distances (Rouzer et al., 2002). Also, the “flattening” of any potential gradient by treating slices cultures with CB1/CB2 agonists had little if any effect on the guidance of neuroblasts in the stream (unpublished observation). We therefore would postulate that 2-AG does not directly impart positional information in the RMS by forming a short or long-range gradient. We favour the alternative hypothesis that autocrine eCB signalling is required for local guidance because it stabilises the leading process (Oudin et al., 2011) allowing the neuroblast to read and/or respond to other *bona fide* guidance cues.

The expression of BDNF and TrkB have been reported throughout the RMS (Chiaramello et al., 2007), however conflicting reports on the requirement of this pathway for migration have appeared. For example, blocking BDNF function with an antibody has been reported to increase both the speed of migration and the number of neuroblasts migrating towards the OB (Bagley and Belluscio, 2010), an observation not readily reconciled with the motogenic function for BDNF on SVZ-derived neuroblasts reported by others (Chiaramello et al., 2007). However, another study found that neuroblast migration in acute brain slices from adult mice is inhibited by incubation with TrkB-Fc (Snapyan et al., 2009). We have revisited this question by determining the effect of a TrkB-Fc on migration in distinct areas of the RMS. Our results show that this treatment clearly disrupts migration; cells spend more time immobile, and when they do move they move slightly slower than control cells in an exploratory rather than directed manner. Non-specific effects of the TrkB-Fc can be excluded based on the observation that a TrkA-Fc had no effect. Our data not only confirm a motogenic role for BDNF (Chiaramello et al., 2007; Snapyan et al., 2009), but also extend its function as a local guidance cue throughout the stream.

The expression of the FGFR1-4 in the SVZ and the localisation of FGF-2 in the SVZ-RMS-OB migration route have been reported on in detail (Garcia-Gonzalez et al., 2010). The FGFR1 is expressed by SVZ derived-neuroblasts, and FGF-2 can stimulate neuroblast migration from RMS explants, however when presented as a soluble molecule there was no evidence for a chemoattractive function (Garcia-Gonzalez et al., 2010). These authors reported high levels of FGF-2 emanating from the SVZ to form a gradient, and postulated that this might drive neuroblasts into the RMS *via* a motogenic mechanism. In the present study we demonstrate that migration at the beginning of the RMS can be severely disrupted by selective FGFR inhibitors and by a FGFR1-Fc that will trap the receptor ligand(s). This was characterised by cells spending more time immobile, and importantly when they did move it was again at speeds similar to the control, but in an exploratory rather than directed manner. We also found that an antibody that neutralises FGF-2 had similar effect as the FGFR inhibitor and the FGFR1-Fc, suggesting that migration is primarily regulated by FGF-2/FGFR1 signalling. If a gradient of FGF-2 emanating from the SVZ provides a “motogenic” drive, migration should be disrupted by flattening the gradient with the exogenous ligand. Interestingly, treatment with FGF-2 did disrupt migration, but like the other agents it affected motility and local guidance.

Importantly, the agents that inhibit FGFR function and perturb migration at the beginning of the stream have no effect on migration at the end of the stream, and this not only provides an exquisite internal control for the specificity of the reagents, but also provides compelling evidence for regional specification of FGFR function. By treating animals with the FGFR inhibitor, we have also provided the first evidence for the FGFR functioning in a region-specific manner in the RMS *in vivo*, with significant decreases in the length of the leading process observed following a 24 h treatment with AZD4547 for neuroblasts sampled towards the beginning but not the end of the stream. Thus we would conclude that the role of the FGFR extends beyond a simple motogenic function in a region-restricted manner in the RMS.

By using live-cell imaging to characterise the role of three distinct receptor systems in the regulation of migration we have been able to identify some additional interesting points. For example, there is no obvious “hierarchy” with the acute disruption of function of any of these, since they have a similar effect on motility and guidance at the beginning of the stream. Using *in vitro* assays, we also show that these pathways do not function in a synergistic manner to drive motility, but are able to function independently from one another. The fact that three receptor systems can function in such a similar manner might provide robustness to the system such that a chronic loss of function in one pathway might be compensated for by adaptation. Another interesting point is that the primary effect on motility is on the length of time the neuroblasts are immobile, rather than on the speed of movement when they do migrate. One possibility is that a maintained momentum might help propel cells towards the bulb with cells losing direction of travel if they become immobile and consequently having to re-explore their immediate environment.

As well as providing novel insights into how the three receptor systems regulate migration, the present study highlights some unresolved issues. In the case of eCB signalling, perhaps the most pertinent question is what actually drives eCB tone in the migratory neuroblast? The FGFR can couple to eCB signalling in neurons to promote axonal growth (Williams et al., 2003), however this pathway cannot be responsible for the eCB migration towards the end of the stream as the FGFR is not functional there. This conclusion is supported by experiments on RMS explant cultures where inhibiting eCB or FGF receptors inhibits migration to a similar extent, and stimulating them promotes migration to a similar extent. The responses were not additive suggesting that they act on a similar pathway. However the pathways are independent of each other at the receptor level as cannabinoid receptors can stimulate migration when FGF receptors are blocked, and FGF-2 can stimulate migration to a certain extent when cannabinoid receptors are blocked. The muted response to FGF-2 in the presence of the cannabinoid block might be due to direct cross-talk between these pathways (Williams et al., 2003). There is also ample evidence for direct cross-talk between TrkB and eCB signalling (Berghuis et al., 2005; Lemtiri-Chlieh and Levine, 2010; Maison et al., 2009) with future studies perhaps warranted to investigate the possibility that they directly interact to promote neuroblast migration in the RMS, given that perturbing each has similar effects throughout the stream.

One of the most interesting results in this study is the direct evidence for a regionally restricted role for FGFR signalling. A gradient of FGF-2 emanating from the SVZ has been reported along the RMS (Garcia-Gonzalez et al., 2010) and matrix-bound gradients of FGF-2 provide positional information in other systems (Wu et al., 2014). Future studies should address how the gradient is formed and maintained within the RMS, and determine if this has a haptotactic function by promoting migration down the gradient and/or inhibiting migration up the gradient. Irrespective of the mechanism, the results of this study provide clear evidence for “long-range” differences in FGFR function within the RMS in living animals and slice cultures, and identify a role for FGF-2 in local guidance that cannot be explained by a simple motogenic function.

In summary, this study provides detailed insights into the consequences of inhibiting eCB, BDNF and FGFR function on neuroblast migration in the intact RMS. It complements recent biochemical and transcriptional profiling studies that have provided insights into the molecular mechanisms that underlie the control of neurogenesis by eCB and FGFR pathways, studies that have identified unique and common pathways (Maccarrone et al., 2014; Sutterlin et al., 2013; Williams et al., 2013) and highlighted the importance of cytoskeletal molecules such as fascin in the regulation of neuroblast migration in the RMS (Sonego et al., 2013a). It also provides direct evidence for FGFR function being required for neuroblast migration within the RMS in a regionally restricted manner.

## 4 Experimental methods

### 4.1 Animals

CD-1 mouse (*Mus musculus*) pups of either sex were used (Charles River, UK). All procedures were performed in accordance with UK Home Office Regulations (Animal Scientific Procedures Act, 1986).

### 4.2 *In vivo* postnatal electroporation

Electroporation of P2/P3 mouse pups was performed as previously described (Oudin et al., 2011; Sonego et al., 2013b). Briefly, mouse pups were anaesthetized with isofluorane (0.6 l/min). Using a pulled glass capillary (diameter 1.5 mm, Clark, UK), 1–2 μl of 1 μg/μl pCX-EGFP plasmid (a kind gift from Dr. Masaru Okabe, Osaka University, Japan) was injected into the right ventricle. Animals were then subjected to five electrical pulses of 99.9 V for 50 ms with 850 ms intervals using the CUY21SC electroporator (Nepagene, Japan) and 5 mm tweezer electrodes (Sonidel, Japan) coated with conductive gel (CEFAR, France). Animals were then reanimated under oxygen and returned to their mother.

### 4.3 Brain slice imaging

Acute brain slice cultures were prepared 5–7 days post-electroporation as previously described (Sonego et al., 2013a). Briefly, electroporated brain hemispheres were cut sagittally in 300 μm-thick slices using a vibratome (VT10005, Leica, Germany). Slices containing GFP-labelled cells in the RMS were cultured on a MilliCell filter membrane (Millipore) for 2 h in phenol red-free DMEM supplemented with 1% glucose, 1% B27, 2% l-glutamine, 10 mM HEPES, 1% Pen/Strep, and 5% FCS (all from Invitrogen) plus selected drugs/factors as described in the text. Slices were then imaged at 37 °C for 3 h using Perkin Elmer UltraView VoX spinning disk system equipped with an inverted Nikon Ti–E microscope using a Nikon CFI Super Plan Fluor ELWD 20X/0.45 objective and a Hamamatsu Orca R2 camera. Frames were taken over a 100–150 μm interval at 2 μm z-steps every 3 min. Images were acquired using the Perkin Elmer Volocity acquisition software.

### 4.4 Imaging data analysis

For analysis of migration in brain slices, we tracked neuroblast migration using the Volocity software as previously described (Sonego et al., 2013a,b). In brief, this generates a value for the distance travelled between two consecutive frames (every 3 min) as well as the total distance travelled over the entire imaging interval (3 h). The displacement (the shortest distance between start and end points) and the average speed for each cell are also automatically measured. The directionality of migration was assessed by calculating the Meandering Index (MI) (*i.e.* the ratio between actual displacement and total distance covered over 3 h). MI values were divided into 3 groups, a method previously used to determine whether cells display *directed* (MI = 0.6–1), *intermediate* (MI is between 0.4–0.6) or *exploratory* (MI = 0–0.4) motility (Nam et al., 2007). Only cells that remained visible in all the frames within the 3 hour imaging period and covered a total distance ≥ 100 μm were included for statistical analysis. The number of time points where the nucleus moved less than 2 μm was considered to calculate the percent time spent as “immobile” by cells. To measure the percent of cells migrating towards the OB, we used a chart generated by the Volocity tracking software, which depicts the tracks for each cell (Sonego et al., 2013a). To obtain the average displacement of each cell population, we calculated the average displacement over a 3 hour period for all the cells within a slice. Within each slice, 20–40 cells within one RMS region were tracked and n = between 6 and 8 animals for all drug conditions. For each treatment we initially analysed cells in the descending arm of the stream before the elbow region (region 1, Fig. 2A), and then proceeded to analyse cells towards the end of the RMS, just before the OB (region 2, Fig. 2A).

For morphological analysis of neuroblasts in brain slices, the length of the leading process of GFP-labelled neuroblasts in fixed brain slices was measured using NIH ImageJ (Das et al., 2014). The ‘elbow’ region of the RMS was used as anatomic marker, and defined as region 2 in Fig. 8. The region after the SVZ and before the ‘elbow’ is defined as region 1; following the ‘elbow’, two regions with same length as region 2 (~ 350 μm) were defined as region 3 and region 4 (Fig. 8). Six consecutive slices were analysed per brain, with at least 100 cells analysed per RMS region. This morphological analysis was carried out in at least 3 animals per condition.

### 4.5 Explant assays

The assay was performed as previously described (Oudin et al., 2011; Ward and Rao, 2005). In brief, RMS explants dissected from P5–P8 mouse pups were embedded in Matrigel (BD Biosciences) diluted 3:1 with fresh Neurobasal medium. When the Matrigel matrix had solidified (after about 15 min at 5% CO2 and 37 °C), medium was added containing various pharmacological reagents or growth factors (see text for details). For RMS explant migration assays, explants were fixed with 4% PFA for 45 min, 6 h or 24 h after embedding in Matrigel. Explants were blocked in 15% goat serum/0.3%Triton-X/phosphate-buffered saline (PBS) for 1 h, and then incubated with Alexa Fluor 488 Phalloidin (1:200, Invitrogen) overnight at 4 °C. Coverslips with the embedded explants were then mounted with Mowiol. Images were taken using an Axiovert 135 microscope equipped with an AxioCam (Zeiss) using a 20×/0.75 objective. To quantify migration out of the RMS explants, pictures were taken on an Apotome microscope (Zeiss). Using ImageJ, we measured the distance between the edge of the explant and the furthest cell (identified by Hoechst staining) for at least 10 different positions around the explant to obtain the average migration distance out of each explant (Falenta et al., 2013). The presented data are obtained from at least 3 independent experiments, with at least 10 explants examined per condition in each experiment.

### 4.6 Pharmacological reagents

The reagents used to inhibit and stimulate eCB signalling are widely used and included a CB1 receptor antagonist (AM251) and agonist (ACEA), a CB2 receptor antagonist (JTE-907) and agonist (JWH-133) (all from Tocris), a monoacyglycerol lipase (MAGL) inhibitor (JZL-184, a gift from Dr Cravatt) and two DAGL inhibitors (OMDM188 and THL, gifts from Dr Di Marzo). They have had their selectivity established in numerous studies (Long et al., 2009; Ortar et al., 2008; Pertwee, 2006) and we have previously reported on their use in migration assays (Oudin et al., 2011) and in some instances on their selective effects on biochemical and transcriptional responses in migratory cells (Sutterlin et al., 2013). Two selective small molecule FGFR inhibitors were used and these were PD173074 (Calbiochem) and AZD4547 (Active Biochem). Their efficacy and selectivity have also been established in numerous studies (Gavine et al., 2012; Mohammadi et al., 1998; Skaper et al., 2000), and in the case of PD173074 also on biochemical and transcriptional responses in migratory cells (Sutterlin et al., 2013). Fusion proteins comprising a human IgG Fc domain and the extracellular domain of a tyrosine kinase receptor are widely used to neutralise the activity of cognate ligands. In this study we have used a TrkA-Fc, TrkB-Fc and FGFR1-Fc (all from R&D) to block their respective ligands. The efficacy and selectivity of these reagents have been widely reported (Binder et al., 1999; Ellsworth et al., 2002). bFM-1 (Millipore) is a very selective monoclonal antibody that blocks FGF-2 signalling (Matsuzaki et al., 1989). In some experiments FGF-2 (R&D) was added to slices or explant cultures.

### 4.7 Drug treatment of live animals and subsequent immunohistochemistry

For morphological analysis of neuroblasts in brain slices from mouse pups treated with drugs, pups were injected intraperitoneally (I.P.) with two doses of AZD4547 (details above) at 12.5 mg/kg 12 h apart, or an equivalent volume of vehicle (DMSO, Sigma) as control 5 days after electroporation. 24 h later, pups were sacrificed. For histological analysis of GFP-labelled neuroblasts, brains were fixed in 4% PFA overnight, embedded in gelatin and cut in 50 μm slices that were immunostained for GFP as previously described (Oudin et al., 2011). Images were taken using a Zeiss LSM 710 confocal microscope equipped with a 40×/1.4 oil DIC objective using the Zen software (Zeiss). Visual inspection of tissue sections stained with Hoechst dye to label nuclei, anti-GFAP antibody to label astrocytes or anti-PSA-NCAM or anti-doublecortin antibodies (DCX) to label migratory neuroblasts from control and drug-treated animals revealed no obvious difference in the general morphology of the RMS or in the expression of these markers following any drug treatment (data not shown).

### 4.8 Statistical analysis

One-way or two-way ANOVA followed by Bonferroni's post-tests were used for all statistical analysis. Where shown, *p < 0.05, **p < 0.01, ***p < 0.001 (all relative to control). For all graphs, the error bars represent the standard error of the mean (s.e.m.).

The following are the supplementary data related to this article.

Supplementary Movie 1 Directed cell migration of a vehicle control movie.
Spinning disc microscopy video of a sagittal mouse brain slices with GFP-labelled neuroblasts. Brain slice was prepared 5 days after *in vivo* postnatal electroporation of P2 mice with pCX-EGFP, cultured with vehicle (DMSO) for 2 h and subsequently imaged every 3 min for 3 h in the same medium. Time-lapse movies made from the descending arm of the RMS with olfactory bulb towards the right bottom corner. The frame rate is 15 frames per second.

Supplementary Movie 2 Disrupted cell migration of a CB1/2 antagonists treated movie.
Spinning disc microscopy video of a sagittal mouse brain slices with GFP-labelled neuroblasts. Brain slice was prepared 5 days after *in vivo* postnatal electroporation of P2 mice with pCX-EGFP, cultured with CB1/2 antagonists AM251 + JTE-907 (both at 1 μM) for 2 h and subsequently imaged every 3 min for 3 h in the same medium. Time-lapse movies made from the descending arm of the RMS with olfactory bulb towards the right bottom corner. The frame rate is 15 frames per second.

Supplementary Movie 3 Disrupted cell migration of a TrkB-Fc treated movie.
Spinning disc microscopy video of a sagittal mouse brain slices with GFP-labelled neuroblasts. Brain slice was prepared 6 days after *in vivo* postnatal electroporation of P2 mice with pCX-EGFP, cultured with TrkB-Fc at 1 μg/ml for 2 h and subsequently imaged every 3 min for 3 h in the same medium. Time-lapse movies made from the descending arm of the RMS with olfactory bulb towards the right bottom corner. The frame rate is 15 frames per second.

Supplementary Movie 4 Disrupted cell migration of a FGFR inhibitor treated movie.
Spinning disc microscopy video of a sagittal mouse brain slices with GFP-labelled neuroblasts. Brain slice was prepared 5 days after *in vivo* postnatal electroporation of P2 mice with pCX-EGFP, cultured with 1 μM AZD4547 for 2 h and subsequently imaged every 3 min for 3 h in the same medium. Time-lapse movies made from the descending arm of the RMS with olfactory bulb towards the right bottom corner. The frame rate is 15 frames per second.

Supplementary Movie 5 Disrupted cell migration of a FGF-2 treated movie.
Spinning disc microscopy video of a sagittal mouse brain slices with GFP-labelled neuroblasts. Brain slice was prepared 5 days after *in vivo* postnatal electroporation of P2 mice with pCX-EGFP, cultured with FGF-2 (50 ng/ml) for 2 h and subsequently imaged every 3 min for 3 h in the same medium. Time-lapse movies made from the descending arm of the RMS with olfactory bulb towards the right bottom corner. The frame rate is 15 frames per second.

Supplementary data to this article can be found online at http://dx.doi.org/10.1016/j.mcn.2014.12.001.

## Competing interests

The authors declare no competing financial interests.

## Author contributions

P.D. Y.Z. and M.J.O. designed the experiments and wrote the paper, G.L. designed the experiments and revised the paper, Y.Z. and M.J.O. performed research and analysed the data, S.G. and M.S. helped in brain slice imaging, K.F. helped with *in vivo* electroporation, G.W. contributed to data analysis methods and provided criticism.

## Funding

The work was supported by the Kings-China PhD studentship (K-CSC) to Y.Z., and by the Wellcome Trust (0892326/Z/09/Z) and BBSRC (D527118/1).
