# Supplementary figures and images for: Regional effects of endocannabinoid, BDNF and FGF receptor signalling on neuroblast motility and guidance along the rostral migratory stream
Source: Mol Cell Neurosci. 2015 Jan;64:32–43. doi: 10.1016/j.mcn.2014.12.001 (PMC4324876; doi:10.1016/j.mcn.2014.12.001)

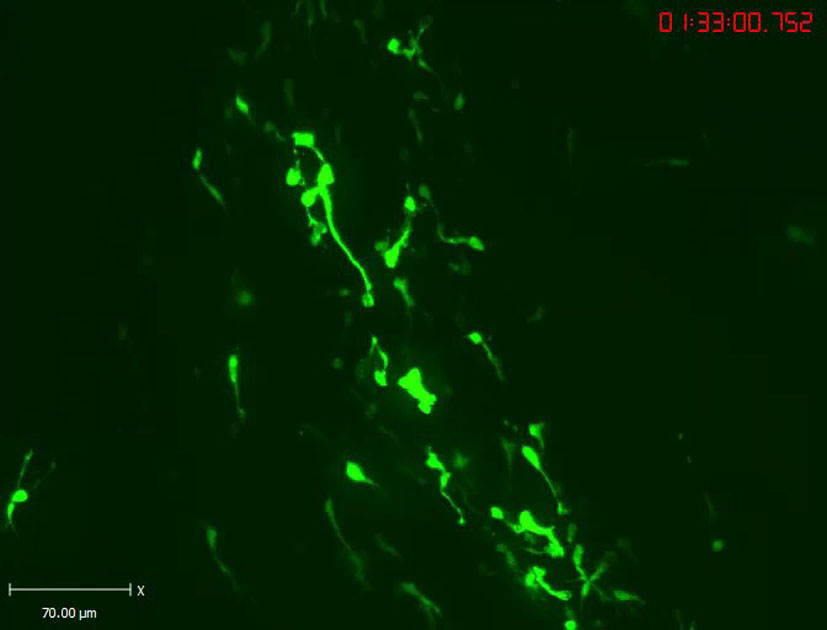

Supplement: Supplementary Movie 1 — Directed cell migration of a vehicle control movie. Spinning disc microscopy video of a sagittal mouse brain slices with GFP-labelled neuroblasts. Brain slice was prepared 5 days after in vivo postnatal electroporation of P2 mice with pCX-EGFP, cultured with vehicle (DMSO) for 2 h and subsequently imaged every 3 min for 3 h in the same medium. Time-lapse movies made from the descending arm of the RMS with olfactory bulb towards the right bottom corner. The frame rate is 15 frames per second. [file mmc1.jpg]

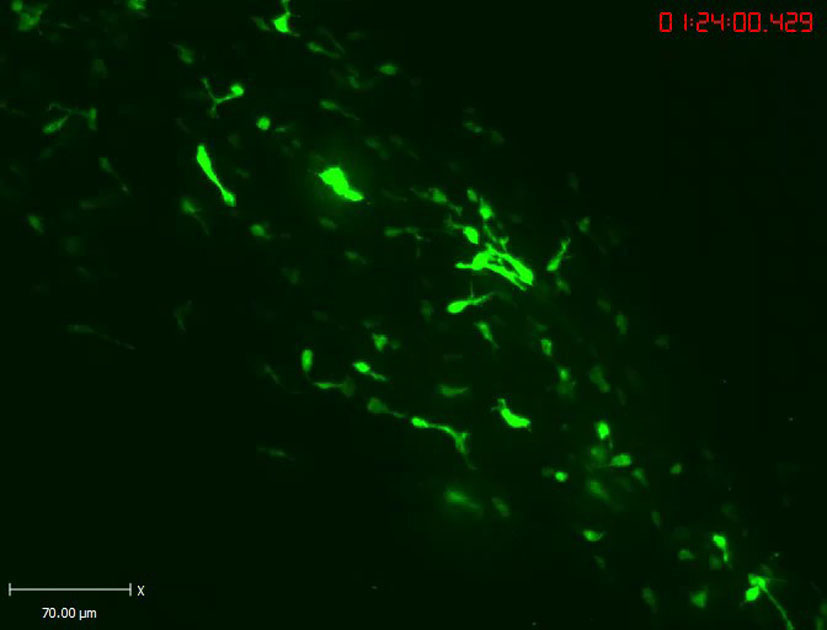

Supplement: Supplementary Movie 2 — Disrupted cell migration of a CB1/2 antagonists treated movie. Spinning disc microscopy video of a sagittal mouse brain slices with GFP-labelled neuroblasts. Brain slice was prepared 5 days after in vivo postnatal electroporation of P2 mice with pCX-EGFP, cultured with CB1/2 antagonists AM251 + JTE-907 (both at 1 μM) for 2 h and subsequently imaged every 3 min for 3 h in the same medium. Time-lapse movies made from the descending arm of the RMS with olfactory bulb towards the right bottom corner. The frame rate is 15 frames per second. [file mmc2.jpg]

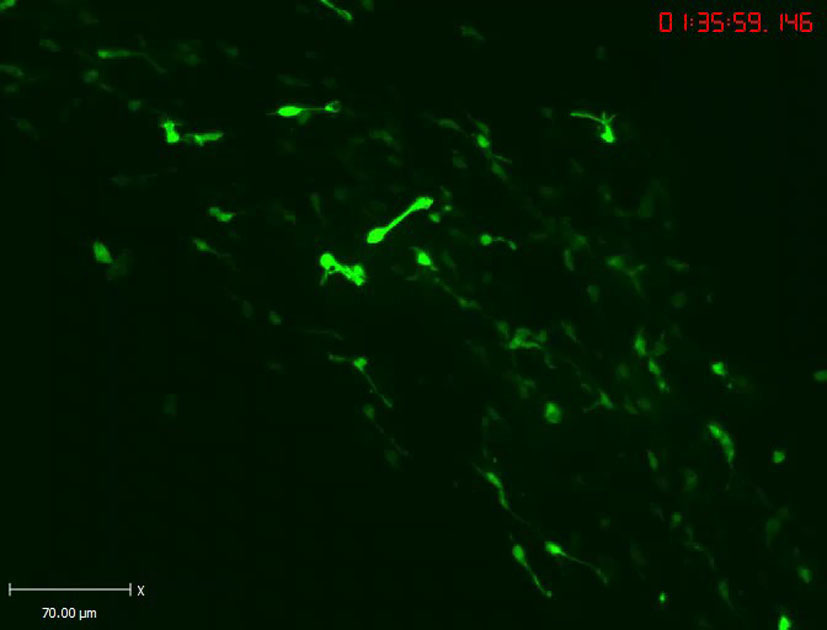

Supplement: Supplementary Movie 3 — Disrupted cell migration of a TrkB-Fc treated movie. Spinning disc microscopy video of a sagittal mouse brain slices with GFP-labelled neuroblasts. Brain slice was prepared 6 days after in vivo postnatal electroporation of P2 mice with pCX-EGFP, cultured with TrkB-Fc at 1 μg/ml for 2 h and subsequently imaged every 3 min for 3 h in the same medium. Time-lapse movies made from the descending arm of the RMS with olfactory bulb towards the right bottom corner. The frame rate is 15 frames per second. [file mmc3.jpg]

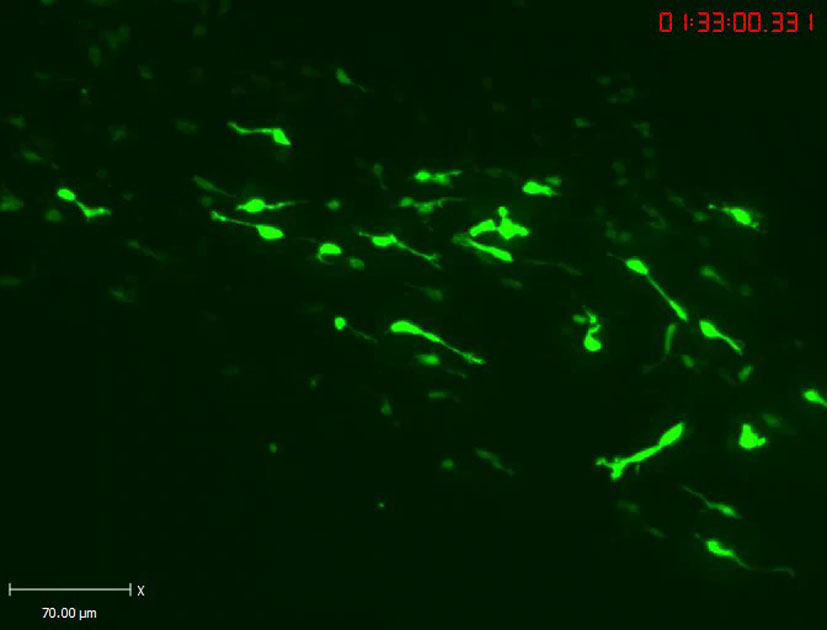

Supplement: Supplementary Movie 4 — Disrupted cell migration of a FGFR inhibitor treated movie. Spinning disc microscopy video of a sagittal mouse brain slices with GFP-labelled neuroblasts. Brain slice was prepared 5 days after in vivo postnatal electroporation of P2 mice with pCX-EGFP, cultured with 1 μM AZD4547 for 2 h and subsequently imaged every 3 min for 3 h in the same medium. Time-lapse movies made from the descending arm of the RMS with olfactory bulb towards the right bottom corner. The frame rate is 15 frames per second. [file mmc4.jpg]

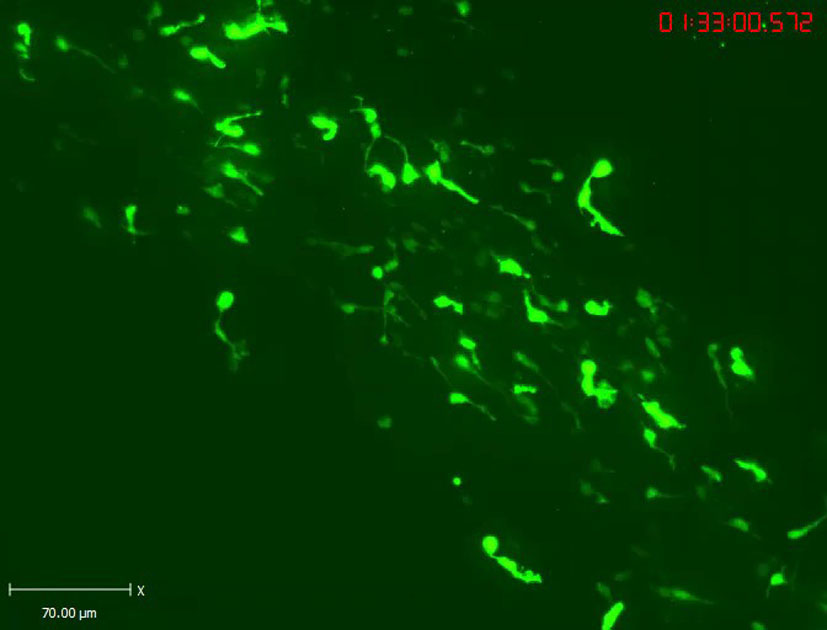

Supplement: Supplementary Movie 5 — Disrupted cell migration of a FGF-2 treated movie. Spinning disc microscopy video of a sagittal mouse brain slices with GFP-labelled neuroblasts. Brain slice was prepared 5 days after in vivo postnatal electroporation of P2 mice with pCX-EGFP, cultured with FGF-2 (50 ng/ml) for 2 h and subsequently imaged every 3 min for 3 h in the same medium. Time-lapse movies made from the descending arm of the RMS with olfactory bulb towards the right bottom corner. The frame rate is 15 frames per second. [file mmc5.jpg]
